# Amplitude dynamics favors synchronization in complex networks

**DOI:** 10.1038/srep24915

**Published:** 2016-04-25

**Authors:** Lucia Valentina Gambuzza, Jesus Gómez-Gardeñes, Mattia Frasca

**Affiliations:** 1 DIEEI, Università degli Studi di Catania, Catania, 95125, Italy; 2 Departamento de Física de la Materia Condensada, Universidad de Zaragoza, E-50009 Zaragoza, Spain; 3 Institute for Biocomputation and Physics of Complex Systems (BIFI), Universidad de Zaragoza, E-50018 Zaragoza, Spain

## Abstract

In this paper we study phase synchronization in random complex networks of coupled periodic oscillators. In particular, we show that, when amplitude dynamics is not negligible, phase synchronization may be enhanced. To illustrate this, we compare the behavior of heterogeneous units with both amplitude and phase dynamics and pure (Kuramoto) phase oscillators. We find that in small network motifs the behavior crucially depends on the topology and on the node frequency distribution. Surprisingly, the microscopic structures for which the amplitude dynamics improves synchronization are those that are statistically more abundant in random complex networks. Thus, amplitude dynamics leads to a general lowering of the synchronization threshold in arbitrary random topologies. Finally, we show that this synchronization enhancement is generic of oscillators close to Hopf bifurcations. To this aim we consider coupled FitzHugh-Nagumo units modeling neuron dynamics.

Synchronization of interacting units is a universal collective behavior appearing in a variety of natural and artificial systems^1^,^2^. The most used mathematical formalisms to study how synchronization shows up, such as the paradigmatic Kuramoto model^3^, consider each dynamical unit as a pure phase oscillator. This coarse-grained dynamical description lies in the following assumption: since each isolated unit has a stable limit cycle oscillation at a given *natural* frequency, it is enough to study the variable accounting for the motion along this limit cycle (the phase), while the other dynamical variables may be ignored. This approach is justified provided the attraction to the limit cycle is strong compared to the coupling between dynamical units^4^. On the contrary, if the coupling is strong or the attraction to the limit cycle is weak, different phenomena, whose analysis demands both amplitude and phase dynamics, such as oscillation death^5^,^6^ and remote synchronization^7^,^8^,^9^, can occur.

The Stuart-Landau (SL) model^10^,^11^ allows to bridge the gap between the simplicity of the Kuramoto model and the completeness of the amplitude-phase frameworks. In particular the SL model may describe, by means of a single parameter, both the behavior close to the Hopf bifurcation, which represents an instance where the attraction to the limit cycle is weak, and the case where, on the contrary, the motion is purely confined to the limit cycle thus behaving as a phase oscillator.

Synchronization of pure phase oscillators interacting according to a complex network topology has been widely studied during the last decade^12^. These studies have covered different topics such as the conditions for the onset of synchronization^13^, the path towards it^14^, the interplay between topology and dynamics^15^,^16^, the emergence of first-order transition^17^,^18^,^19^,^20^, the effect of noise on the robustness of the global state^21^, among others. However, interesting issues about the interplay of a network topology of interactions and the coupled dynamics of the amplitude and phase of the oscillators are often ignored from scratch, due to the underlying dynamical framework chosen.

In this paper, we adopt the framework of SL oscillators coupled according to different network configurations and compare with its reduction to pure phase oscillators. Our goal is to investigate the effect of the amplitude dynamics near the Hopf bifurcation on the synchronization in random complex networks. The most striking result is that, when the amplitude dynamics is not negligible and the natural oscillation frequencies of the nodes are not homogeneous, synchronization is enhanced regardless of the topology of the underlying network. The result holds for oscillators close to the Hopf bifurcation and in particular we also discuss its implications for neuron-like dynamics, by illustrating the behavior for the FitzHugh-Nagumo model.

## Model

Our analysis is based on a system of *N* coupled SL oscillators formally described by:


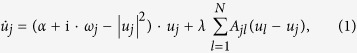


where *u*_*j*_ is a complex variable so that in polar coordinates: 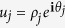, being *ρ*_*j*_ the amplitude of oscillator *j* and *θ*_*j*_ its phase. *α* is the Hopf bifurcation parameter which controls how fast the trajectory decays onto the attractor, *ω*_*j*_ is the frequency of oscillator *j* when uncoupled (natural frequency), *λ* the coupling strength between interacting units and *A*_*jl*_ is the network adjacency matrix (defined as *A*_*jl*_ = *A*_*lj*_ = 1 if *j* and *l* are connected, *A*_*jl*_ = 0 otherwise).

In this work, system (1) is studied with respect to the parameters *α* and *λ* and different frequency distributions. Importantly, when *α* is large compared to coupling *λ*, the amplitude dynamics vanishes, *i.e.*, 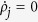 and 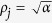. In this limiting case Eq. (1) reduce to:


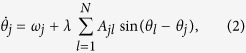


*i.e.*, the Kuramoto model in a network. As introduced above, our aim is to compare phase synchronization in networks of amplitude and phase oscillators with that in networks of pure phase oscillators. In doing so, we unveil the key role of the parameter ruling the Hopf bifurcation, which transforms a generic oscillator into a pure phase one driving the system far from the critical point. To this aim, we consider SL oscillators [Eq. (1)] with small *α* (in particular, *α* = 1), while for Kuramoto (K) oscillators we study Eq. (2) or, equivalently, Eq. (1) with large *α*. We will then discuss the universality of our findings by studying dynamics other than the SL in the proximity of the Hopf bifurcation.

Phase synchronization in the model is monitored through the order parameter *r*. This parameter, rigorously defined in the Methods section, represents the degree of global synchronization in the whole network. Values of *r* close to 1 indicates that all the pairs of oscillators are phase synchronized, whereas *r* = 0 in the absence of synchronization.

## Results

### Motifs

We start our discussion by considering the onset of synchronization in small undirected network motifs. In particular we analyze the 2-node motif (representing a pair of coupled oscillators) and two 3-node motifs (the open and the closed triangle). In the case of the open triangle we have three different configurations depending on how natural frequencies are assigned to each of the three oscillators. In particular, if we indicate as *ω*_1_ the natural frequency of the degree-2 (central) node we have: *i*) *ω*_1_ > *ω*_2_, *ω*_3_; *ii*) *ω*_1_ < *ω*_2_, *ω*_3_; and *iii*) *ω*_2_ < *ω*_1_ < *ω*_3_ (or equivalently *ω*_2_ > *ω*_1_ > *ω*_3_).

**Figure 1:**
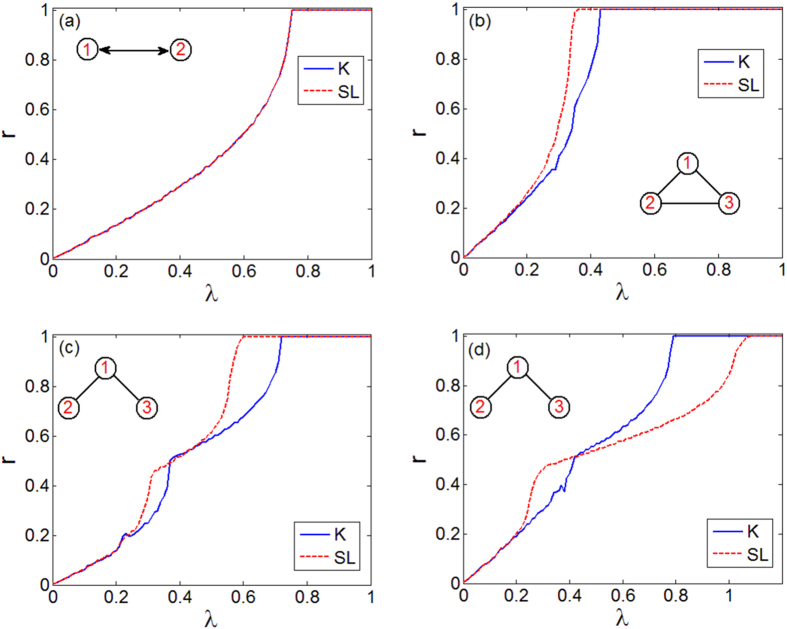
Synchronization for a 2-node motif and 3-node motifs of Kuramoto and Stuart-Landau oscillators: (**a**) 2-node motif with *ω*_1_ = 2.5 and *ω*_2_ = 1.01; (**b**) closed triangle with *ω*_1_ = 2.51, *ω*_2_ = 1.01, *ω*_3_ = 1.66; (**c**) open triangle with *ω*_1_ = 2.51, *ω*_2_ = 1.01, *ω*_3_ = 1.66; (**d**) open triangle with *ω*_1_ = 1.66, *ω*_2_ = 1.01, *ω*_3_ = 2.51.

To illustrate the possible scenarios, in Fig. 1 we show the evolution of the degree of synchronization *r* as a function of the coupling *λ* for both the SL and K dynamics in 4 network motifs for a specific set of frequency values. By indicating as 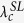 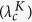 the value of the coupling for which the SL (K) oscillators reach full synchronization, *i.e. r* = 1, we observe that, in the 2-node motif [Fig. 1(a)], 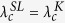, so the two types of oscillators display no difference in the synchronization level. On the contrary, in the closed triangle [Fig. 1(b)] 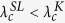, *i.e.*, the transition to full synchronization in the SL oscillators occurs for a lower coupling than in the K oscillators. Finally, in the case of the open triangle motif we observe either 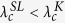 [Fig. 1(c)] or 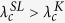 [Fig. 1(d)], depending on the distribution of the natural frequencies of the oscillators.

**Figure 2:**
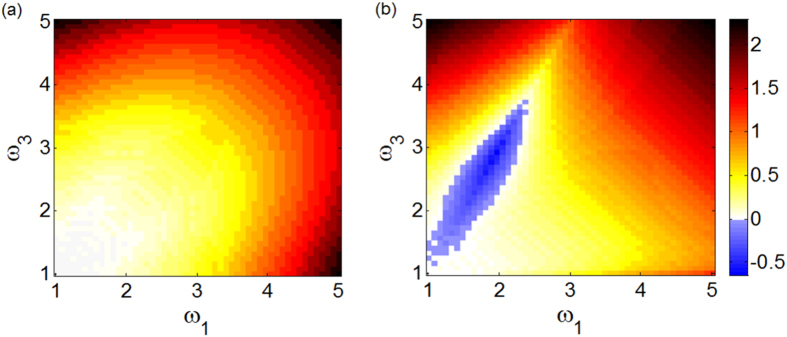
Behavior of 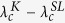 (color coded) as a function of *ω*_1_ and *ω*_3_ for *ω*_2_ = 1.01 in the closed (**a**) and open (**b**) triangle. For the closed triangle 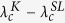 is always positive, indicating an enhancement of synchronization occurring in this motif for SL oscillators, independently from the frequency distribution. For the open triangle, an enhancement is found independently from the specific values of the frequencies, only provided that *ω*_1_ > *ω*_2_, *ω*_3_ (or *ω*_1_ < *ω*_2_, *ω*_3_, data not shown).

To characterize how the system behavior is influenced by the natural frequencies, without loss of generality we have fixed *ω*_2_ = 1.01 and systematically varied *ω*_1_ and *ω*_3_ in [1.01, 5] for the closed and open triangle. For each pair (*ω*_1_, *ω*_3_), we calculate 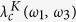 and 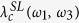. Figure 2 illustrates the results: the color codes account for the difference between the two synchronization thresholds, that is, 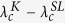. For the closed triangle (Fig. 2(a)), 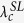 is always lower than 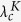. On the other hand, for the open triangle (Fig. 2(b)), 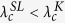 in the region below the main diagonal (where *ω*_1_ > *ω*_3_), whereas in the region above the main diagonal (where *ω*_3_ > *ω*_1_) the sign of 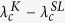 may be positive or negative. Given the symmetry of the structure, we derive that:

- When *ω*_1_ > *ω*_2_, *ω*_3_ (or *ω*_1_ < *ω*_2_, *ω*_3_), 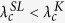 both in the open and closed triangles.
- When *ω*_2_ < *ω*_1_ < *ω*_3_ (or *ω*_2_ > *ω*_1_ > *ω*_3_), 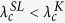 for the closed triangle, while this does not always hold for the open one.

Hence, in the open triangle the critical value of the coupling is lower in SL oscillators than in K oscillators, when the natural frequency of the central oscillator (1) is either larger or smaller than those of the two peripheral units (2 and 3), while, otherwise, we can observe either 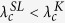 or 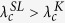.

As a conclusion, the dynamical behavior of the 3-node motifs leads to the identification of three types of configurations: the closed triangle, which we indicate as motif A, the open triangle with either *ω*_1_ > *ω*_2_, *ω*_3_ or *ω*_1_ < *ω*_2_, *ω*_3_ (motif B), and the open triangle with either *ω*_2_ < *ω*_1_ < *ω*_3_ or *ω*_3_ < *ω*_1_ < *ω*_2_ (motif C). Interestingly, the presence of an unconstrained amplitude variable yields an enhancing of synchronization in both motifs A and B with respect to the usual K framework.

We now tackle the stability analysis of the open/closed triangle to gain insight about the behavior observed numerically. Note that the case of a pair of coupled oscillators has been already analytically treated for both K^1^ and SL^4^ oscillators, yielding 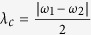. We first rewrite Eq. (1) in polar coordinates for the triangle motif, and define *φ*_12_ = *θ*_1_ − *θ*_2_ and *φ*_13_ = *θ*_1_ − *θ*_3_ to obtain:


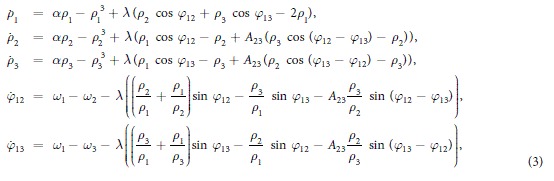


where *A*_23_ = 1 (*A*_23_ = 0) in the closed (open) triangle. Eq. (3) are written in compact form as 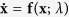 with **x** = [*ρ*_1_, *ρ*_2_, *ρ*_3_, *φ*_12_, *φ*_13_]^*T*^, where we have highlighted the dependence on the parameter *λ* as we perform our analysis with respect to different values of the coupling. The fully synchronized state, denoted as 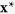, implies stationary amplitudes and constant phase differences. Therefore, we compute the solution of 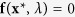 (system 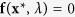 admits more than one solution, as it is symmetric with respect to *ρ*_*i*_, however, only one solution has positive amplitudes) and, then, study the stability of the solution by inspecting the sign of the eigenvalues of the Jacobian matrix 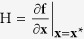.

**Figure 3:**
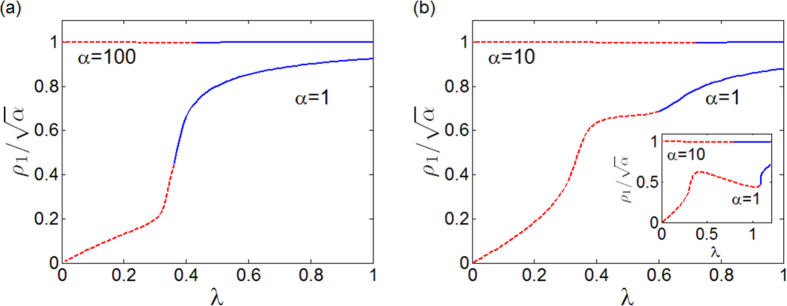
Stability of the solution of Eq. (3) for two different values of *α* (*α* = 1, SL and *α* = 100, K oscillators) and two motifs: (**a**) closed triangle; (**b**) open triangle. Dashed (continuous) line indicates unstable (stable) solutions. The natural frequencies are fixed as *ω*_1_ = 2.51, *ω*_2_ = 1.01, *ω*_3_ = 1.66 (and as *ω*_1_ = 1.66, *ω*_2_ = 1.01, *ω*_3_ = 2.51 in the inset).

The results for the closed and open triangle are illustrated in Fig. 3 by showing the equilibrium value for the first component 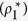 of 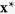 for *α* = 1, representative of the behavior of amplitude and phase oscillators, and *α* = 100, representative of the limiting K case when the attraction to the limit cycle is strong. The first result observed from Fig. 3 is the confirmation that for *α* = 100 the amplitude, independently of the value of *λ*, is always constrained to its value on the limit cycle 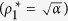. More importantly, the exact value of *λ*_*c*_ is represented as the boundary between the unstable (dashed lines) and stable (continuous lines) solutions. As shown, for the closed triangle [Fig. 3(a)] the transition to stability for *α* = 1 occurs at a lower value than for *α* = 100, while for the open triangle [Fig. 3(b)] this depends on the frequency distribution: in the main panel of Fig. 3(b) 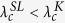, while in the inset of Fig. 3(b) the opposite holds. Thus, the stability analysis confirms, as shown numerically in Fig. 1, that amplitude dynamics favors synchronization in motifs A and B, while in motif C we can find scenarios where it does not promote synchronization.

### Networks

We now show that amplitude dynamics, in the proximity of the Hopf bifurcation, may favor phase synchronization in large populations of heterogeneous oscillators coupled through random network topologies. To illustrate this, we consider two paradigmatic examples of network topologies, scale-free (SF) and Erdös-Rényi (ER) networks (see Methods). The results reported below are obtained in ER and SF networks of *N* = 500 nodes, but their consistency has been checked for larger *N*, up to *N* = 2000. Without loss of generality, the natural frequencies of the oscillators, {*ω*_*j*_}, are uniformly distributed in the interval [1, 3]. However, once *N* natural frequencies are randomly generated, they are assigned to the *N* nodes following one of these two different frameworks: *(i)* the frequencies are randomly assigned (RA) to the nodes, or *(ii)* the frequencies are first ordered in descending order and then assigned to the nodes from the highest degree one to the lowest (ordered assignment, OA). Orderings based on other centrality measures can be also considered. However, as the networks investigated here are undirected and unweighted, measures of centrality based on node degree, eigenvector centrality, page rank and betweenness, are strongly correlated each other, thus yielding to almost indistinguishable results.

**Figure 4:**
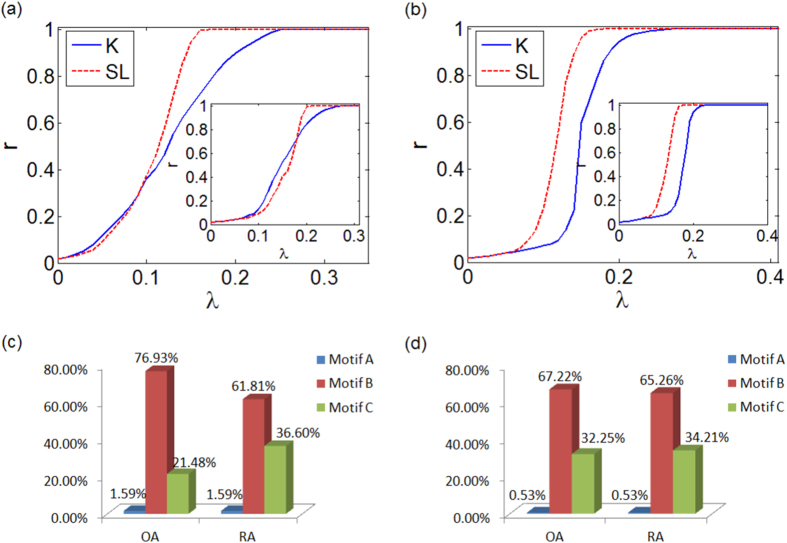
Synchronization for a network of K and SL oscillators: order parameter *r* vs. *λ* for a SF topology (**a**) and an ER topology (**b**); occurrence of motifs for the SF network (**c**) and the ER network (**d**). The main panels of (**a**,**b**) refer to networks with a frequency-node assignment ruled by OA, while the insets to RA. Networks have *N* = 500 and mean degree 〈*k*〉 = 8.

In Fig. 4 we show *r*(*λ*) for SF [Fig. 4(a)] and ER [Fig. 4(b)] networks. The main panels illustrate the OA case, while the insets the RA one. Remarkably, in all cases, we observe that 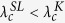 so that synchronization is enhanced for SL oscillators with respect to K ones, being this effect more evident when OA applies.

We now link the macroscopic behavior observed in Fig. 4(a,b) to the microscopic structure of networks. In fact, a large network may be decomposed into elementary blocks, that is, the motifs^22^, and it is known that they have a role in the synchronization properties of the network^23^,^24^. We consider the 3-node motifs A, B and C, and, in order to illustrate their effect on the global behavior of the system, we study their occurrence in the networks analyzed. The results of our analysis are shown in Fig. 4(c,d) for both OA and RA frameworks. Obviously, due to the low clustering of SF and ER networks, the occurrence of closed triangles (motif A) is relatively low and thus the enhancement of synchronization in SL oscillators cannot be explained by the presence of such microscopic structure. Instead, such enhancement seems to be rooted in the abundance of motif B with respect to C in both ER and SF networks, since (at a microscopic scale) amplitude dynamics benefits from motif B to reach synchronized states while the presence of motif C could be detrimental for the synchronization of SL oscillators.

The occurrence of motifs clearly depends on two factors: the topology of the network and the way in which the natural frequencies are assigned to the nodes. In particular, the number of closed (motif A) or open triangles (motifs B and C) is only determined by the network topology, while the algorithm for the frequency-node assignment is the responsible for the differences between the occurrences of motifs B and C. For instance, in the case of OA it is clear that motif B is favored over motif C as OA assigns larger frequencies to higher degree nodes. Thus, in the case of open triangles, OA automatically sets *ω*_1_ > *ω*_2_, *ω*_3_.

Contrary to what can be expected at a first glance, RA also favors the occurrence of motif B over motif C, thus producing an enhancement of synchronization in networks of SL oscillators. In this case the reasons behind the under-representation of motif C are purely combinatorial. Consider the open triangle and three frequencies *ω*_*a*_, *ω*_*b*_ and *ω*_*c*_ such that *ω*_*a*_ < *ω*_*b*_ < *ω*_*c*_. The six possible ways to distribute *ω*_*a*_, *ω*_*b*_ and *ω*_*c*_ to the three nodes labeled as 1 (the central one), 2 and 3 lead to four motifs of type B and two of class C. Hence, as in the case of OA, in a random network with the natural frequencies assigned according to RA, the higher occurrence of motif B with respect to motif C, results in the enhancement of synchronization when the oscillators have their amplitude as a free variable (SL case).

**Figure 5:**
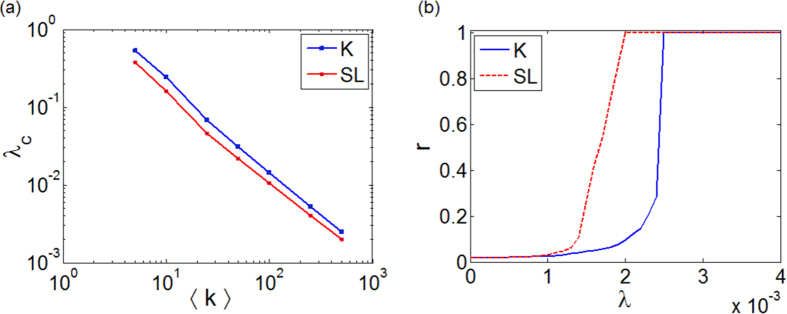
Effect of the connectivity in ER networks on 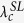 and 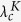 (**a**) and order parameter *r* vs. *λ* for a fully connected (all-to-all) topology (**b**). The network size is *N* = 500.

We have so far considered networks with relatively low connectivity, 〈*k*〉 = 8. As the mean connectivity increases one expects that the number of closed triangles (motif A) grows while the ratio between the occurrence of motif B and C remains unaltered. Thus, considering that motif A also acts as a synchronization promoter, one expects a further synchronization enhancement as 〈*k*〉 increases. This effect is shown in Fig. 5(a), which reports 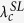 and 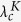 for random ER networks with increasing values of the mean degree 〈*k*〉. Importantly, the curve of 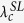 is always below that of 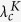. Figure 5(b) illustrates the full connectivity case, *i.e.* the all-to-all topology, confirming that synchronization enhancement due to the amplitude dynamics also holds in this asymptotic case. Thus, synchronization enhancement due to the amplitude dynamics is also evident in networks with higher connectivity, including the full connectivity case. Additionally, Fig. 5(a) also shows that both thresholds decrease with the connectivity. This is consistent with Fig. 1(b–d) where it can be seen that both 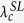 and 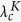 are lower for closed than for open triangles. We also note that in Fig. 5(a) the clustering coefficient increases with 〈*k*〉, leading to an enhancement of synchronization. However, a large increase of clustering implies the appearance of higher order network motifs, thus demanding a study about the role that such fundamental structures play in the global behavior of the system.

**Figure 6:**
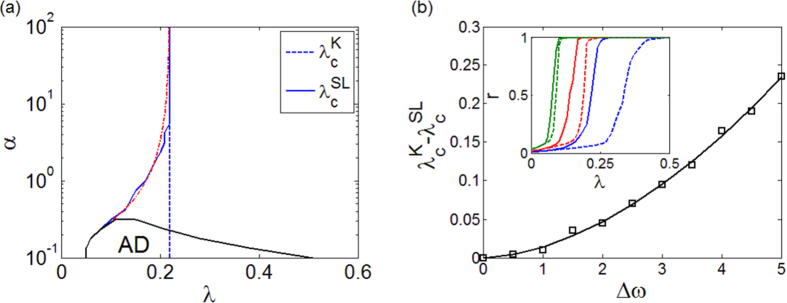
(**a**) Critical values 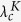 (dashed line), 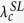 (solid line) and fitting of 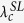 (red, dash-dot line) in the plane *λ* − *α*. AD indicates the region of amplitude death. (**b**) 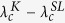 as a function of Δ*ω*, showing how larger heterogeneities increase the gap between the two thresholds. The inset illustrates the order parameter *r* for SL (continuous line) and K (dashed line) oscillators for three different values of Δ*ω* (Δ*ω* = 1 green line, Δ*ω* = 2 red line and Δ*ω* = 4 blue line). Data refer to an ER network with *N* = 500 and 〈*k*〉 = 8, where the frequencies are assigned according to RA.

To round off, we now explore the effects that two dynamical ingredients, *α* and the frequency heterogeneity, play in the onset of synchronized states. Up to now, our analysis has been performed by focusing on a fixed value of *α*, that is, *α* = 1 (*α* = 100) for the SL (K) oscillator, and a uniform distribution of natural frequencies within a fixed interval (*ω*_*j*_ ∈ [1, 3]). However, the critical value 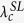 depends on both factors, and, in particular, it decreases with *α, i.e.*, as the amplitude dynamics becomes more relevant. This result becomes clear in Fig. 6(a) where the curves of 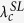 (solid line) and of 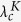 (dashed line) are reported in the plane *λ* − *α* for an ER network (similar results are obtained for motifs and SF networks). When *α* is large, the behavior of SL oscillators is similar to that of K ones, and the curve of 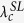 superimposes to that of 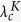. On the contrary, the smaller becomes *α*, the closer are SL oscillators to the Hopf bifurcation, a condition that favors synchronization. In particular, Fig. 6(a) shows that, soon after *α* = 10 the critical couplings 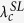 and 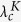 become different, so that synchronization is significantly favored for SL oscillators (outside the region of amplitude death). The threshold gap 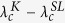 is fitted by 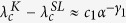 with *c*_1_ = 0.048 ± 0.005, *γ*_1_ = 0.76 ± 0.1. The fitting is also reported in Fig. 6(a).

We have also studied the behavior with respect to distributions in [1, 1 + Δ*ω*] and observed that larger values of Δ*ω* further promote the enhancement of synchronization in networks of SL oscillators with respect to those with K units. In particular, Fig. 6(b) illustrates 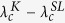 vs. Δ*ω*. The curve is functionally fitted by 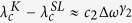 with *c*_2_ = 0.014 ± 0.0025 and *γ*_2_ = 1.75 ± 0.12. The parameter Δ*ω* quantifies the degree of heterogeneity in the node dynamics. Increasing Δ*ω* makes more difficult to achieve synchronization in the network, thus generally increasing the synchronization threshold (for the case of two oscillators, the analytical formula 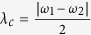 clearly shows this). However, the increase in the threshold for the Kuramoto oscillators is greater than for SL, thus resulting in the larger gap 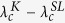 observed. This positive effect of heterogeneity is also found in the motifs (see Fig. 2), where the greater is the difference between the oscillator frequencies the larger is the gap between the two thresholds.

## Discussion

Since the seminal paper by Rosenblum, Pikovsky and Kurths^25^, it is well established that amplitude affects synchronization. In fact, in a general framework, the phase of an oscillator (chaotic or periodic) may be written^26^ as:


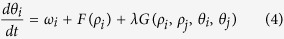


where 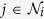 and 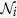 is the set of first neighbors of *i*. If the oscillator is chaotic, then in phase synchronization amplitudes are not correlated and the contribution *F*(*ρ*_*i*_) may be viewed as a stochastic term^26^. As noise can enhance synchronization, then, it could be argued that ultimately the phenomenon reported in our work is due to the beneficial presence of noisy signals. However, in the case of Stuart-Landau equations, Eq. (4) can be explicitly written as:


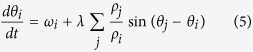


from where it is recognized that


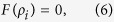



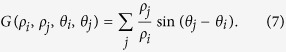


As *F*(*ρ*_*i*_) = 0, the observed phenomenon cannot be ascribed to an effect of noise terms. Instead, it is rooted in the dependence of the coupling function, *G*(*ρ*_*i*_, *ρ*_*j*_, *θ*_*i*_, *θ*_*j*_), on the amplitude and, moreover, is clearly a network effect as it does not appear for a pair of oscillators (Fig. 1(a)). The dependence of the coupling on the amplitude is not trivial and generates the behavior that is captured from the study of the full system (Eq. 1) performed through the stability analysis of its solutions. In fact, when the system synchronizes the amplitudes assume stationary values which depend on the model parameters (*λ, α* and frequency distribution). In particular, a stronger contribution of amplitude variations is obtained for oscillators closer to the Hopf bifurcation, as controlled by the parameter *α*. This is a generic mechanism that we have illustrated with SL equations, representing the normal form of Hopf bifurcation^27^. For this reason we expect that our findings are universal and are observed in other systems near the Hopf bifurcation.

**Figure 7:**
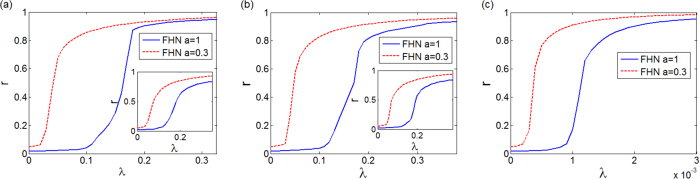
Synchronization for a network of FHN oscillators with *N* = 500. Order parameter *r* vs. *λ* for: (**a**) SF topology, (**b**) ER topology, (**c**) fully connected (all-to-all) topology. The main panels of (**a**,**b**) refer to networks with a frequency-node assignment ruled by OA, while the insets to RA. The parameters have been set as: *ε* = 0.01; *b* = 0.2; *ω*_*i*_ such that the natural oscillation frequencies are randomly distributed (with uniform distribution) in the interval [1, 3]; *a* is set to *a* = 0.3 (red line) or to *a* = 1 (blue line) to study oscillators at different distances from the Hopf bifurcation occurring at *a*_*c*_ = 0.

To verify whether the phenomenon is generic, we have analyzed the FitzHugh-Nagumo (FHN) equations, which are able to capture the spiking dynamics of neurons and serve as fundamental model of excitable behavior in neural communication^28^,^29^. We have performed numerical simulations on *N* coupled FHN units:


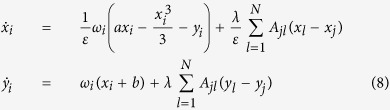


where *ε, a, b, ω*_*i*_ are constant parameters. In particular, *ω*_*i*_ sets the frequency of oscillation of the unit *i* when uncoupled from the others and *a* controls the distance to the critical Hopf bifurcation point (*a*_*c*_ = 0). We have analyzed system (8) under conditions similar to those defined for the study of the SL oscillators, starting from 3-node motifs to larger networks, and found consistent results. For example, Fig. 7 illustrates the order parameter *r* calculated for SF, ER and fully connected (all-to-all) topologies, showing that oscillators closer to the bifurcation (curve for *a* = 0.3) have a synchronization threshold lower than those far from the critical point (curve for *a* = 1).

Summarizing, the study of synchronization in networks of oscillators is often carried out by assuming that the motion is described by a phase variable only or, equivalently, that the attraction to the limit cycle is strong compared to the coupling strength. However, we have shown that, when this simplification is dropped, phase synchronization is enhanced. This important result holds for generic systems near the Hopf bifurcation and therefore may be relevant for neuroscience, nanoscale resonators, electronic circuits, and many other fields where synchronization arises in a spontaneous or intentionally induced way. The result also appears to be quite robust under changes of macroscopic structural features of the networks, such as their degree heterogeneity, as it is rooted in the dynamical behavior of the microscopic building blocks of the network. Thus, our results show that, despite of the simplicity of limit cycle oscillators, new effects are to be observed in the synchronization of complex networks when important dynamical features, such as amplitude dynamics, are taken into account.

## Methods

### Numerical simulations

Eqs. (1) are integrated with a 4th order Runge-Kutta algorithm with fixed step size Δ*t* = 0.01 for *T*_*s*_ = 1000, and Eq. (8) with Δ*t* = 0.001 for *T*_*s*_ = 500.

### Synchronization order parameter

To quantify the degree of phase synchronization of a pair of network oscillators, *j* and *l*, the time averaged order parameter 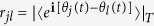, where *θ*_*j*_(*t*) is the phase variable of oscillator *j* at time *t*, and 〈·〉_*T*_ is the average over the time interval *T*, is considered. Furthermore, the degree of global synchronization in the whole network is measured through the parameter 
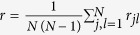
, which takes values close to 1 when all the pairs of oscillators are phase synchronized, whereas *r* = 0 in the absence of synchronization. Provided that the time interval *T* is large enough, the measure is robust to changes in it. In our simulations, we have set *T* = *T*_*s*_/2.

### Networks’ construction

We consider Erdos-Renyi (ER) networks, that is, networks having a connectivity distribution (the probability that a given node has a given number of links) following a Poisson-like distribution, and scale-free (SF) networks, that is, networks with a heterogeneous degree distribution (a power-law distribution). Both networks are generated by means of the model introduced in ref. 30 that allows to control the mean connectivity, 〈*k*〉, in order to be exactly the same. The model has a single tunable parameter which allows to interpolate between ER and SF networks as far as the degree distribution is concerned. Networks with *N* = 500 nodes have been used, and the consistency of results has been checked for larger *N* up to *N* = 2000.

## Additional Information

**How to cite this article**: Gambuzza, L. V. *et al*. Amplitude dynamics favors synchronization in complex networks. *Sci. Rep.* **6**, 24915; doi: 10.1038/srep24915 (2016).
